# Vertebrate Host Susceptibility to Heartland Virus

**DOI:** 10.3201/eid2212.160472

**Published:** 2016-12

**Authors:** Angela M. Bosco-Lauth, Amanda E. Calvert, J. Jeffrey Root, Tom Gidlewski, Brian H. Bird, Richard A. Bowen, Atis Muehlenbachs, Sherif R. Zaki, Aaron C. Brault

**Affiliations:** Centers for Disease Control and Prevention, Fort Collins, Colorado, USA (A.M. Bosco-Lauth, A.E. Calvert, A.C. Brault);; Colorado State University; Fort Collins (A.M. Bosco-Lauth, R.A. Bowen);; US Department of Agriculture, Fort Collins (J.J. Root, T. Gidlewski);; Centers for Disease Control and Prevention, Atlanta, Georgia, USA (B.H. Bird, A. Muehlenbachs, S.R. Zaki)

**Keywords:** Heartland virus, HRTV, phlebovirus, viruses, vertebrates, mice, Ag129 mice, mouse model, chickens, hamsters, goats, rabbits, raccoons, host susceptibility, virulence, vector-borne infections, zoonoses

## Abstract

Heartland virus (HRTV) is a recently described phlebovirus initially isolated in 2009 from 2 humans who had leukopenia and thrombocytopenia. Serologic assessment of domestic and wild animal populations near the residence of 1 of these persons showed high exposure rates to raccoons, white-tailed deer, and horses. To our knowledge, no laboratory-based assessments of viremic potential of animals infected with HRTV have been performed. We experimentally inoculated several vertebrates (raccoons, goats, chickens, rabbits, hamsters, C57BL/6 mice, and interferon-α/β/γ receptor–deficient [Ag129]) mice with this virus. All animals showed immune responses against HRTV after primary or secondary exposure. However, neutralizing antibody responses were limited. Only Ag129 mice showed detectable viremia and associated illness and death, which were dose dependent. Ag129 mice also showed development of mean peak viral antibody titers >8 log_10_ PFU/mL, hemorrhagic hepatic lesions, splenomegaly, and large amounts of HRTV antigen in mononuclear cells and hematopoietic cells in the spleen.

## Abstract 2

Virus-infected Ag129 mice could be a useful model for identifying tick infection or virus transmission.

Heartland virus (HRTV) is a novel bunyavirus (family *Bunyaviridae*, genus *Phlebovirus*) first identified in the United States in 2 persons with thrombocytopenia/leukopenia in Missouri in 2009 (*1*). The virus has subsequently been isolated from nymphal *Amblyomma americanum* ticks collected at sites adjacent to the residences of the index case-patients (*2*). High seroprevalence rates for HRTV have been reported in northern raccoons (*Procyon lotor*), white-tailed deer (*Odocoileus virginianus*), and horses sampled near the residences of human case-patients (*3*). Additional serosurveillance in wildlife has demonstrated wide-ranging antibody prevalence to HRTV across the geographic host range distribution of *A. americanum* ticks (*4*).

Genetically, HRTV is most closely related to severe fever with thrombocytopenia syndrome virus (SFTSV) (*1*), a tickborne virus in Asia that causes high fever, thrombocytopenia, leukopenia, gastrointestinal disorders, central nervous system involvement, disseminated intravascular coagulopathy; and multiorgan dysfunction. Infection with this virus shows a case-fatality rate of ≈12% in humans (*5*,*6*). Severe disease progression of patients infected with SFTSV has further been associated with increased cytokine and chemokine responses (*7*) and increased viremias throughout the course of disease (*6*). Infection of rhesus macaques with SFTSV caused mild human disease syndromes similar to those in humans, including thrombocytopenia, leukopenia, and increased levels of hepatic and cardiac enzymes (*8*).

Unlike SFTSV, which has been associated with thousands of cases of symptomatic human disease in China (*5*) and South Korea (*9*), only 8 diagnosed human cases of HRTV infection have been reported in the United States, and 1 death in 2013 (*10*). Of those patients, 7 were from Missouri and 1 from Tennessee (this patient died), which indicated the continued presence of the virus over the course of several years and over a wide geographic range (*11*). Similar to that described for SFTSV, patients infected with HRTV commonly have fever, fatigue, anorexia, thrombocytopenia, leukopenia, and increased levels of hepatic enzymes (*11*).

With the recent discovery of SFTSV, HRTV, and other novel phleboviral agents in recent years (*12*–*14*), it is apparent that tickborne phleboviruses have been largely underrecognized as potential human disease agents. The vertebrate host range associations for SFTSV have been poorly described and have not been addressed for HRTV by experimental animal inoculation studies.

Given the serologic evidence that northern raccoons are commonly exposed to HRTV across the central and eastern United States (*4*) and near the residence of a human index case-patient (*3*), we performed experimental inoculations of raccoons with HRTV. In addition, on the basis of results of goat serosurveillance for SFTSV in China (*15*) and experimental infections (*16*), we performed experimental inoculation of goats with HRTV. We also assessed animal models (chickens, rabbits, hamsters, and mice) for host competence for HRTV to identify host range restrictions of the virus and establish a potential model for viremic blood feeding for subsequent tick infections.

## Materials and Methods

### Viruses and Animal Models

We used an isolate of HRTV (Mo4) obtained from serum of an acutely ill human patient in the fall of 2009 for experimental infections and plaque reduction neutralization tests (PRNTs). The isolate was passaged once on Vero E6 cells (African green monkey kidney cell line). Lone Star virus (LSV) strain 2229 was used as a control virus for intraperitoneal and intracranial inoculation of CD-1 mice. All animal models (including strain, age, inoculation route, and dose inoculated) assessed for host competence for HRTV are shown in Table 1. New Zealand white rabbits, chickens, Syrian golden hamsters, C57BL/6 mice, and CD-1 mice were obtained from Charles River Laboratories (Wilmington, MA, USA) and housed at the Centers for Disease Control and Prevention (Fort Collins, CO, USA) in Animal Biosafety Level 3 (ABSL 3) conditions. Ag129 mice (α/β/γ receptor knockout) (*17*) were originally obtained from B & K Universal (Hull, UK) and bred in house. The receptor knockout genotype of the Ag129 mouse breeding line was confirmed by genetic PCR markers generated from tail snip tissues from 10 mice (Transnetyx, Cordova, TN, USA).

**Table 1:** Experimental inoculation of vertebrate hosts with Heartland virus*

Experimental animal model	Strain	No.	Age, d/sex	Inoculum dose, PFU	Route of inoculation
Mouse	C57BL/6	15	21/ F	10^4^	ip
Mouse	Ag129	15	21/M and F	10^4^	ip
Mouse	Ag129	15	21/M and F	10^3^	ip
Mouse	Ag129	15	21/M and F	10^2^	ip
Mouse	Ag129	15	21/M and F	10^1^	ip
Mouse	Ag129	15	21/M and F	10^0^	ip
Mouse†	CD-1	10	2	10^3^	ic
Mouse†	CD-1	10	17	10^3^	ip
Chicken	Leghorn	3	Adult/F	10^4^	sc
Hamster	Syrian golden	5	21/F	10^4^	sc
Goat	Boer	2	Adult/M and F	10^4^	sc
Rabbit	New Zealand white	3	Adult/F	10^4^	sc
Raccoon	Wild-caught	6	Adult/M and F	10^4^	sc

*Ag129, interferon-α/β/γ receptor–deficient; ic, intracranial; ip, intraperitoneal; sc, subcutaneous. †CD-1 mice were also inoculated similarly with Lone Star virus (same dose and route as for Heartland virus).

Boer goats were obtained from a private vendor and housed in the Colorado State University Animal Disease ABSL 3 Laboratory. Six raccoons were caught in the wild in Larimer County, Colorado, and housed in a US Department of Agriculture facility for several months before being transferred to the Animal Disease ABSL 3 Laboratory at Colorado State University for experimental inoculation. Raccoon serum samples were screened for neutralizing antibodies against HRTV/phlebovirus as described (*3*) and in this report. All animal handling, trapping, and care was in compliance with approved Institutional Animal Care and Use Committee protocols under the oversight of attending veterinarians. At arrival in the laboratory, all animals were housed for a minimum of 3 days for acclimatization before inoculation.

### Experimental Inoculations

We inoculated animals with 10^0^–10^5^ PFU by subcutaneous, intracranial, or intraperitoneal routes (Table 1). Whole blood was collected from individual hamsters, rabbits, chickens and goats daily during 1–7 days postinoculation (dpi), groups of 3 raccoons on alternating days 1–8 dpi, and 3 groups of 5 or 6 C57BL/6 and Ag129 mice every third day 1–9 dpi. Ag129 mice were divided into 5 dosage groups of 15 mice (10^0^–10^4^ PFU) (Table 1). At 28 dpi, animals from all groups (except raccoons) were bled and then given booster inoculations with 10^4^ PFU of HRTV and analyzed through 42 dpi, at which point the animals were bled and euthanized. Terminal blood samples were obtained from mice by cardiac bleed under deep isoflurane anesthesia just before euthanasia. Two Ag129 mice (10^2^ PFU dose group) were terminally bled at 4 days postsecondary challenge, and spleens were harvested for production of monoclonal antibodies (MAbs) as described (*18*).

Raccoons were anesthetized with a ketamine:xylazine cocktail (15–20 mg/kg:3–4 mg/kg) (*3*) given by intramuscular inoculation before sampling, and mice and hamsters were anesthetized in an isoflurane chamber before serum sampling. Rabbits, chickens, and goats were manually restrained appropriately for sample collection without use of anesthetics. Whole blood was collected from peripheral saphenous, cheek, or tail veins (mouse and hamster); ear (rabbit); or jugular (chicken and raccoon). Samples were centrifuged, and serum samples were frozen for subsequent virologic or serologic evaluation. Relative neuroinvasive and neurovirulence phenotypes for HRTV were compared with that for LSV strain 2229 by intraperitoneal and intracranial inoculation, respectively. Isoflurane-anesthetized suckling (2 days old) and weanling (17 days old) CD-1 mice (n = 10) were inoculated with a 20 μL of sterile phosphate-buffered saline (PBS) suspension of 10^3^ PFU of either virus. A group inoculated with minimal essential medium was included as an inoculation control for both routes of inoculation.

### Serologic Testing

HRTV neutralizing and total immune reactive antibodies were quantified by using a 70% PRNT (PRNT_70_) (*18*) and ELISA, respectively. For PRNTs, serum samples were heat-inactivated at 56°C for 30 min, and 2-fold serially diluted serum samples were mixed with an equal volume of virus suspension. Plaques were enumerated through 10 dpi, and 70% neutralization endpoints were calculated on the basis of comparison with serum negative controls as described (*3*).

ELISAs were performed in 96-well microtiter plates (Immulon 2HB; Thermo Lab Systems, Franklin, MA, USA). Plate wells were coated with purified virus (0.06 μg/well) in buffer (50 mmol/L sodium carbonate, 50 mmol/L sodium bicarbonate, pH 9.6) and incubated at 4°C overnight (*18*). Plates were washed 5 times with a PBS/0.1% Tween wash buffer, and nonspecific binding sites were blocked with Starting Block (Pierce, Dallas, TX, USA) (100 μL/well). Serum samples were added in serial dilutions in PBS (50 μL/well) and incubated at 37°C for 1 h. Plates were washed 5 times before addition of the species-appropriate horseradish peroxidase conjugate (50 μL/well), diluted 1:5,000 in PBS. After an incubation period of 1 h at 37°C, plates were washed and enhanced K-Blue TMB (3,3′,5,5′ tetramethylbenzidine) substrate (Neogen, Lexington, KY, USA) was added to each well (100 μL/well). The plates were incubated in the dark at room temperature for 10 min. Optical density (OD) was read on an automatic plate reader at 450 nm. Endpoints were determined as an OD reading on duplicate samples that was at least twice that of the average OD for control serum samples. Titers were expressed as the geometric mean of the reciprocal of the endpoints.

### Immunohistochemical Analysis

HRTV-inoculated Ag129 mice with signs of disease were subjected to necropsy. Spleen, liver, and kidney tissues were removed, fixed in 10% neutral-buffered formalin, and processed by using routine methods (*10*). Immunohistochemical analysis using rabbit polyclonal antisera against HRTV nucleocapsid (N) protein and an immuno-alkaline phosphatase detection system with Fast Red Chromogen (Biocare Medical, Concord, CA, USA) was used as described (*1*).

## Results

### Viremia, Death, and Clinical Assessment

Serum samples from chickens, raccoons, goats, rabbits, and hamsters obtained during 1–7 dpi did not show detectable HRTV viremia (Table 2). Despite the previous finding that SFTSV replicates to detectable levels in serum and results in pathologic changes in spleens of immunocompetent C57BL/6 mice (*19*), viremia was not detected in any serum samples from this strain of mice inoculated with HRTV. No infection-associated deaths or clinical signs of illness were observed throughout the course of the infection in any of inoculated animals.

**Table 2:** Viremia and antibody responses of animals experimentally inoculated with Heartland virus*

Animal	Inoculum dose, PFU	Mean peak titer†	dpi	Mean ELISA titer‡	ELISA positive, no. (%)	Mean PRNT_70_ titer‡	PRNT_70_ positive, no. (%)
Mouse (C57BL/6)	10^4^	<1.5	42§	4.4 (0.6)	15 (94)	0.7 (0.6)	10 (63)
**Chicken**	**10^4^**	**<1.5**	**14**	**NT**	**NA**	**ND**	**0**
Chicken	10^4^	NA	42§	3.0 (0.2)	3 (100)	ND	0
Hamster	10^4^	<1.5	42§	4.3 (0.5)	4 (100)	1.3 (0.4)	5 (100)
**Goat**	**10^5^**	**<1.5**	**28**	**NT**	**NA**	**1.0**	**2 (100)**
Goat	10^5^	NA	42§	3.0	2 (100)	1.3	2 (100)
**Rabbit**	**10^4^**	**<1.5**	**14**	**NT**	**NA**	**ND**	**0**
Rabbit	10^4^	NA	42§	4.1	3 (100)	1.2 (0.2)	3 (100)
Raccoon	10^4^	<1.5	42	NT	NA	0.4 (0.6)	2 (33)

*Bold indicates initial (not given a booster immunization) sampling of a pair of animals that were later given a booster immunization. dpi, day postinoculation; NA, not assessed; ND, not detected (no titer <1:10 for any animal); NT, not tested; PRNT_70_, 70% plaque reduction neutralization test. †Detection limit was 1.5 log_10_ PFU/mL. ‡log_10_ reciprocal titer (SD).  §Animals were given booster inoculations with 10^4^ PFU of Heartland virus at 28 dpi.

**Figure 1:**
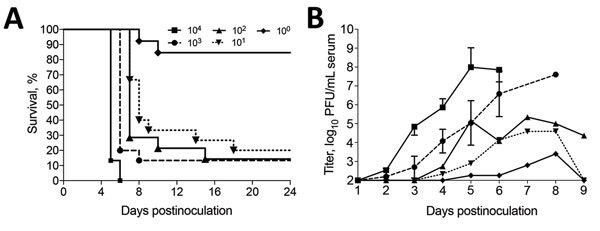
Dose response of Heartland virus (HRTV)–infected interferon α/β/γ receptor–deficient (Ag129) mice. Mice of either sex were inoculated with 10^4^–10° PFU of HRTV/0.1 mL of inoculum. Mice were observed daily for death through day postinoculation 24. A) Percentage survival. B) Dose-associated HRTV viremias determined by plaque assays on Vero E6 cells. Different groups of 5 mice inoculated with the same dose of HRTV were bled every third day. Thus, a decrease in viremia was observed for the 10^2^ PFU dose inoculum group days postinoculation 5 and 6. Error bars indicate SD.

In contrast, 100% of CD-1 mice inoculated intracranially with LSV died within 6 dpi. However, illness or death were not observed in mice inoculated with minimal essential medium or HRTV. Similar to intraperitoneally inoculated C57BL/6 mice, intraperitoneally inoculated CD-1 mice showed no signs of illness or death. However, Ag129 mice showed virus dose-dependent death. Deaths were observed in the group inoculated with 10^4^ PFU; the first signs of illness appeared on 4 dpi and death occurred on 5 dpi (Figure 1, panel A). Groups inoculated with 10^3^, 10^2^, 10^1^, or 10^0^ PFU had mortality rates of 85%, 83%, 80%, and 20%, respectively. Average survival time was inversely proportional to the inoculation dosage. Mean ± SD survival time ranged from 4.1 ± 0.4 d for the 10^4^ PFU dose group to 19.4 ± 4.2 d for the 10^1^ PFU dose group (Figure 1, panel A). Mean ± SD time of death also showed prolonged survival rates with lower inoculation doses when survivors were omitted from the analyses: 4.1 ± 0.4 d (10^4^), 5.1 ± 0.3 d (10^3^), 7.7 ± 2.2 d (10^2^), 9.1 ± 3.7 d (10^1^), and 9 ± 1.4 d (10^0^) (Figure 1, panel A). We calculated a 50% lethal dose of 9 PFU by using linear regression analysis of the mortality rate at various viral dilutions (R^2^ = 0.84).

Viremia titers were also dose dependent; peak titers were highest in the 10^4^ PFU group and lowest in the 10^0^ PFU group (Table 3). Onset of viremia in most of the inoculation groups was at 2 dpi or 3 dpi, and the 10^4^ PFU inoculation group had the highest initial daily viremia titer at 2 dpi (2.5 ± 0.5 log_10_ PFU/mL serum) and the highest mean daily peak viremias by 5 dpi (8 log_10_ PFU/mL serum) (Figure 1, panel B). However, 3 of the mice had viremias at 5 dpi >9 log_10_ PFU/mL serum. Only 5 of 15 Ag129 mice in the 10^0^ inoculation group had detectable viremias; highest detectable titer was 4 log_10_ PFU/mL serum. Clinical signs in Ag129 mice included hunched back, ruffled fur, mucopurulent ocular discharge, and hematochezia. Mice that had these signs were euthanized and subjected to necropsy for gross and histopathologic analysis of lesions.

**Table 3:** Viremia and antibody responses of interferon-α/β/γ receptor–deficient mice experimentally inoculated with Heartland virus*

Inoculum dose, PFU	dpi	Mean peak titer†	Mean ELISA titer	ELISA positive, no. (%)	Mean PRNT_70_ titer‡	PRNT_70_ positive, no. (%)
**10^4^**	**NA**	**8 (1.0)**	**NA**	−	**NA**	−
10^4^	NA	−	NA	**−**	NA	**−**
**10^3^**	**21**	**6.6 (1.2)**	**4.1 (2.0)**	**1 (50)**	**NT**	−
**10^2^**	**28**	**5.3 (2.3)**	**3.9 (1.7)**	**1 (50)**	**NT**	**NT**
10^2^	32	−	NT	**−**	2.2 (0.4)	2(100)§
**10^1^**	**28**	**4.6 (2.1)**	**3.5 (1.4)**	**1 (33)**	**1.5 (0.6)**	**ND**
10^1^	42	−	NT	**−**	1.3 (0.5)	1 (33)
**10^0^**	**28**	**3.4 (2.2)**	**2.7 (1.4)**	**1 (9)**	**1**	**1 (9)**
10^0^	42	−	NT	**−**	1.5 (0.6)	2 (33)

*Bold indicates initial (not given a booster immunization) sampling of a pair of animals that were later given a booster immunization. dpi, day postinoculation; NA, not applicable because uniform deaths were observed; ND, not detected; NT, not tested; PRNT_70_, 70% plaque reduction neutralization test; −, not assessed for titer. †Peak titers (log_10_ PFU/mL of serum) (SD). Detection limit was 1.5 log_10_ PFU/mL. ‡log_10_ reciprocal titer (SD). §Tested at 4 d postchallenge.

### Serologic Analysis

All animals except raccoons received booster inoculations at 28 dpi with 10^4^ PFU of HRTV. HRTV neutralizing antibodies were not detected in chickens through 42 dpi after booster inoculation, but ELISA titers were evident (3.0 log_10_) (Table 2). Low, but detectable, PRNT_70_ titers (1 log_10_–1.3 log_10_) were observed in rabbits given booster inoculations; ELISA titers were <4.1 log_10_. All hamsters given booster inoculations had neutralizing titers (1 log_10_–1.9 log_10_) and ELISA titers (4.3 log_10_). Despite the high HRTV neutralizing antibody prevalence and magnitude of the immune response identified in the field for raccoons, 0 of 6 experimentally inoculated raccoons had detectable viremia, and only 2 had PRNT_70_ titers (1 log_10_ and 1.3 log_10_). ELISA titers for raccoons were not assessed because of a lack of appropriate species-associated conjugate. The 2 goats had low (1.3 log_10_) neutralizing immune responses after booster inoculation, and ELISA titers of 3.0 log_10_ were detected. C57BL/6 mice responded to infection with no detectable (0.7 log_10_) titers detected by PRNT and 4.4 log_10_ titers detected by ELISA (Table 2).

### Serologic Response and Challenge of Surviving Ag129 Mice

Eighteen (24%) of 75 Ag129 mice survived the initial inoculation with HRTV (11 from the 10^0^ PFU group, 3 from the 10^1^ PFU group, 2 from the 10^2^ PFU group, and 2 from the 10^3^ PFU group). The 2 surviving Ag129 mice from the 10^3^ PFU group were terminally bled for immune responses at 21 dpi. One mouse had an ELISA titer of 1:364,500, and the other mouse had no detectable reactivity (titer <1:500) (Table 3). The 2 Ag129 mice that survived the 10^2^ PFU inoculation dose had ELISA titers of 1:121,500 and <1:500 at 28 dpi. These mice were given booster inoculations with 10^4^ PFU of HRTV, and spleens harvested 4 days later (32 dpi) for detection of splenocyte fusions and myeloma cells for MAb development as described (*18*). At 32 dpi (4 days post-HRTV booster inoculation/challenge), neutralizing responses were detected in both mice (1.9 log_10_ and 2.5 log_10_ reciprocal titers), and no serum viremia or virus was detected in the spleens.

Only 1 of the 3 Ag129 mice that survived initial challenge with HRTV at a dose of 10^1^ PFU showed immune reactivity to HRTV antigen by ELISA (titer 1:121,500) at 28 dpi. Similarly, 1 of 11 surviving Ag129 mice from the 10^0^ PFU dose group showed HRTV immune reactivity (titer 1:1,500) (Table 3) at 28 dpi. An HRTV neutralizing titer of 1:10 was identified in the 28 dpi serum sample from the ELISA-positive surviving mouse from the 10^0^ PFU inoculation group. In addition, the surviving mouse with the higher ELISA titer from the 10^1^ PFU dose group did not have a detectable PRNT_70_ titer, although neutralizing titers were observed at lower thresholds. These 14 surviving mice were challenged with 10^4^ PFU of HRTV at 28 dpi and observed for an additional 14 days through 42 dpi. Eight (57%) of the 14 secondarily challenged Ag129 mice survived through 42 dpi (2/3 in the 10^1^ PFU dose group and 6/11 in the 10^0^ PFU dose group), which indicated a protective immune response. However, only 3 of the 8 (1 in 10^1^ PFU dose group and 2 in 10^0^ PFU dose group) surviving mice had detectable neutralizing antibodies against HRTV, and the mean ± PRNT_70_ titers were only 1.9 and 1.5 ± 0.6 for the 2 dose groups, respectively (Table 3).

### Pathologic Analysis

**Figure 2:**
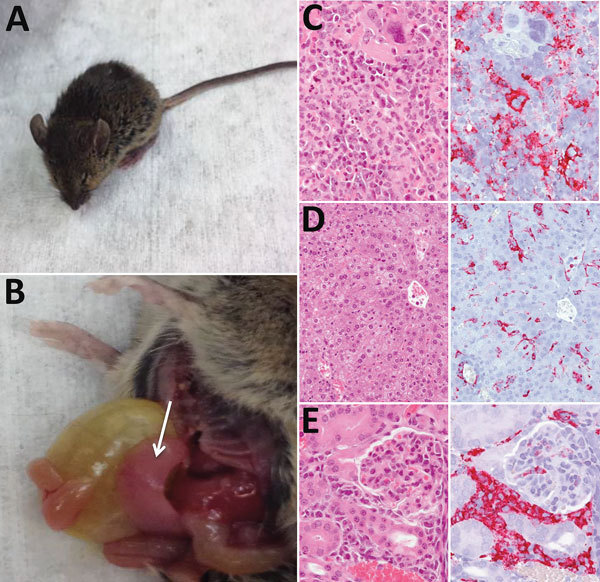
Pathologic changes associated with infection of interferon-α/β/γ receptor–deficient (Ag129) mice with Heartland virus (HRTV). A) Mouse showing typical clinical signs of HRTV infection (ruffled fur, hunched posture, and squinting eyes). B) Dissected mouse showing an enlarged pale spleen (arrow). C–E) Hematoxylin and eosin staining (left panels) and immunohistochemical staining (right panels) for HRTV nucleocapsid protein of spleen (C), liver (D), and kidney (E) of Ag129 mice at 5–7 days postinoculation with 10^4^ PFU virus. Original magnifications: C, ×100; D, ×50; E, ×100.

At necropsy, hamsters, chickens, rabbits, raccoons, goats, and C57BL/6 mice were in good physical condition and none showed gross pathologic lesions associated with HRTV infection. In contrast, Ag129 mice had clear signs of illness (Figure 2, panel A), including hunched posture, ruffled fur, rectal hemorrhage, weight loss, and conjunctivitis. Euthanized Ag129 mice had grossly enlarged, pale spleens, hepatic hemorrhage, enlarged gall bladders, and excess peritoneal fluid (Figure 2, panel B). Hematoxylin and eosin–stained sections of Ag129 spleen and liver and tissues showed reactive white pulp (Figure 2, panel C), abundant apoptotic debris and hepatic sinusoids (Figure 2, panel D), and increased numbers of mononuclear cells. Immunohistochemical analysis showed abundant HRTV antigen was observed diffusely within splenic mononuclear cells (Figure 2, panel C), hepatic sinusoidal (Kupffer) cells (Figure 2, panel D), and renal interstitial mononuclear cells (Figure 2, panel E).

## Discussion

HRTV is a novel bunyavirus known to cause serious disease in a small number of humans. Serologic surveillance and isolation from *A. americanum* tick nymphs suggested that HRTV circulates in an enzootic cycle and spills over into humans when they are exposed to infected ticks (*2*,*3*). However, in the animal models we present in this study, no evidence of replicative infection in any of the wildlife or outbred animals tested was observed, and viremia, illness, and death could only be induced in type I and type II interferon-receptor knockout mice. Likewise, although SFTSV has been found to cause viremia and pathologic manifestations, such as leukopenia and thrombocytopenia in C57BL/6 mice, which is consistent with human pathologic changes, these mice did not show any clinical signs of disease, and evidence of viral replication was limited to the spleen (*19*).

However, all interferon α/β receptor knockout mice (IFNAR −/−) inoculated with SFTSV died within 3–4 dpi and showed marked SFTSV antigen expression and increased viremia profiles than did wild-type mice of the same genetic background (*19*,*20*), which is consistent with our results for Ag129 mice experimentally inoculated with HTRV (*20*). Furthermore, the protective role of the interferon response has also been demonstrated by depleting interferon in age-resistant C57BL/6 mice infected with another phlebovirus (Punta Toro virus) (*21*). These data and the experimental inoculation data in our study indicate that the interferon response is critical for controlling pathologic changes of mice infected with phleboviruses.

Similar to pathologic changes reported for SFTSV infection in C57BL/6 mice (*19*) and IFNAR knockout mice (*20*), viremia and major pathologic changes developed in Ag129 mice inoculated with HTRV, which is consistent with platelet depletion and subsequent thrombocytopenia. Histopathologic analysis showed a tropism for mononuclear cells, as shown by detection of HRTV N protein in splenic mononuclear cells, Kupffer cells, and interstitial mononuclear cells within the kidney. Future studies are needed to directly assess the capacity of the Ag129 mouse model to recapitulate leukopenia and thrombocytopenia manifestations observed in human disease progression and the subsequent role of splenic macrophages with platelet depletion (*19*).

Nine hydridomas expressing HRTV MAbs derived from HRTV-inoculated Ag129 splenocyte fusions described in this report have been characterized. All of them were found to be nonneutralizing and reactive to linear epitopes of the HRTV N protein (*18*). These results are not surprising because phlebovirus N protein is the most abundant protein in the virion and in virus-infected cells (*22*–*24*). Similarly, most MAbs against SFTSV are also specific for N protein and nonneutralizing (*25*,*26*). Because the humoral response to N protein occurs early in infection, this protein is used as a diagnostic antigen (*22*,*27*–*30*). The presence of N protein-targeted humoral responses, measured by ELISA titers, in C57BL/6 and Ag129 mice with low neutralizing activity supports the hypothesis that the N protein could serve as an immunologic decoy. Poor humoral neutralizing response of infected hosts to another bunyavirus, Crimean-Congo hemorrhagic fever virus (genus *Nairovirus*), has been associated with enhanced strain-specific virulence in humans (*31*). Crimean-Congo hemorrhagic fever virus is rapidly cleared in immunocompetent mice, replicates to high titer, and induces pathologic changes and thrombocytopenia in IFNAR mice, similar to the disparate results obtained in this experiment between C57BL/6 and Ag129 mice (*22*,*27*–*30*).

The lack of proper receptors, such as dendritic cell–specific intercellular adhesion molecule 3–grabbing nonintegrin (DC-SIGN) on Vero cells, used in neutralization assays could also explain the low neutralization titers we observed. When compared with cells that were modified to express DC-SIGN, Mukherjee et al. found that neutralization titers to dengue virus were found to be increased (*32*). DC-SIGN has been shown to serve as a receptor for attachment and endocytosis in phleboviral infections, including those with SFTSV, Rift Valley fever virus, and Uukuniemi virus (*33*,*34*). The extremely low level of detected neutralizing antibodies in Ag129 mice that subsequently survived challenge with 1,000 50% lethal doses of HRTV indicates that neutralizing antibody assays are not congruent with humoral protection levels or that cell-mediated immune responses afford considerable protection.

The vertebrate host competence studies reported here also demonstrate the potential host specificity that could have evolved over long periods concomitant with diversification of HRTV from other phleboviruses. Our findings do not exclude the potential role of multiple vertebrate host(s) for maintenance of HRTV; several key animals, such as horses and white-tailed deer, for which there is serologic evidence of field exposures and tick associations, have yet to be assessed for HRTV host competence. Nymphal *A. americanum* ticks, from which HRTV has been isolated (*2*), are known to be catholic feeders, and white-tailed deer may be a major host for the nymphal stage of these ticks (*35*). HRTV could potentially circulate without the need for actual replication in the vertebrate host through direct tick-to-tick transmission while co-feeding nearby on a vertebrate host, as described for the orthomyxovirus, Thogoto virus (*36*).

The role of direct tick transmission of virus to the vertebrate host should be considered because it is related to modulation of the host innate immune response and the potential establishment of a permissive environment for HRTV replication. Previous studies have demonstrated that salivary transmission of Thogoto virus has potentiated the antagonism of interferon-induced Mx1 antiviral activity and resulted in successful transmission of co-feeding ticks on a mouse strain for which parenteral inoculation was unsuccessful (*37*). Salivary transmission by ticks could be a major factor because of immunomodulatory effects of the saliva (*38*,*39*) or differential immune signaling because of specific lectins incorporated onto viral structural proteins, which result from growth in arthropod cells (*40*). This modulation by salivary components could affect initial replication in skin dendritic cells and result in subsequent development of viremias and neutralizing titers in field-collected animals. Repeated exposure of attached ticks salivating virus over prolonged periods could be sufficient for neutralization titers to be manifested in the absence of viral replication.

In conclusion, this study provides an assessment of the relative susceptibility of several vertebrate hosts to parenteral infection with HRTV and a preliminary assessment of a potential model for HRTV-associated disease pathology in humans. Ag129 mice could be used as a source for establishing direct tick infection or as a transmission model for infected *A. americanum* ticks for subsequent assessment of these and alternative natural vertebrate host candidates for HRTV transmission.
